# Study on the measurement of coupling and coordinated development level between China’s internet and elderly care services and its influencing factors

**DOI:** 10.1186/s12889-024-18291-6

**Published:** 2024-03-29

**Authors:** Hu Yangming, Li Sha, Liu Hui, Yang Yanda

**Affiliations:** 1 College of Public Administration and Law, Hunan Agricultural University, Changsha, People’s Republic of China; 2 Animal Science and Technology College, Hunan Agricultural University, Changsha, People’s Republic of China

**Keywords:** Internet, Elderly care services, Entropy method, Coupling coordination degree model, Tobit model

## Abstract

**Background:**

With the intensification of China’s aging population, the demand for elderly care services has become increasingly prominent. At the same time, rapid development of internet technology provides more convenience and possibilities for the elderly. However, the coordinated development between the internet and elderly care services still faces challenges. This study aims to measure the level of coupling and coordinated development between the internet and elderly care services in China, and analyze the influencing factors, in order to provide reference for promoting elderly care services.

**Methods:**

In this paper, the entropy method and coupling coordination degree model were used to measure the coupling coordination development index of the internet and elderly care services in China from 2012 to 2021. In addition, considering that the coordinated development between the two is affected by many factors, the Tobit model was used to analyze the main factors affecting the integration of the internet and elderly care services.

**Results:**

(1) The coupling and coordination of the Internet and senior care services is in its infancy, but the coupling and coordination of the two is on the rise, and there is still a lot of room for development in the future. (2) In terms of time scale, the coupling coordination development level between the internet and elderly care services in China has gone through three stages of “disorder recession-transition coordination-coordinated development”. (3) In terms of influencing factors, government management ability has a more positive impact on the development of the integration of the Internet and senior care services, financial support, scientific and technological investment and the level of innovation play a mild pulling role, while the level of informatization to a certain extent restricts the level of integration of the Internet and senior care services.

**Conclusion:**

In order to promote the coordinated development of China’s Internet and senior care services, it is necessary to comprehensively understand the current situation and development space of China’s Internet and senior care services coupling coordination degree, accurately grasp the dynamic trend of China’s Internet and senior care services coupling and coordinated development, promote the stage of leapfrogging, and fully consider the influencing factors, so as to realize the optimal allocation of policies and resources. These measures will help to promote a more coordinated and sustainable development of the internet and elderly care services in China.

**Supplementary Information:**

The online version contains supplementary material available at 10.1186/s12889-024-18291-6.

## Background

As of 2021, China’s elderly population aged 60 and above has reached 264 million, accounting for 18.7% of the total population [1], and China has become an aging society. As China enters an aging society, the elderly population is growing rapidly, and the demand for elderly care services is becoming increasingly strong. The traditional elderly care model can no longer meet the diversified and efficient needs of modern society, and the rise of Internet technology has brought unprecedented opportunities for elderly care services [2]. Therefore, it is necessary to explore the coordinated development of the Internet and elderly services in this context. The purpose of this study is to measure the level of coupled and coordinated development of Internet and senior care services in China by using the entropy value method, coupled coordination degree model, and Tobit model, and to analyze its influencing factors.

In recent years, academia has conducted extensive research on the combination of the Internet and elderly care services, which can be mainly categorized into two aspects: the promotion of elderly care service development by Internet technology and the driving force for Internet technology innovation through the demands of elderly care services. The role of Internet technology in promoting elderly care services is manifested in several aspects. Firstly, the development of internet technology has brought innovative solutions to elderly care services. Through the integration of smart technology, network connectivity, and data analysis, the internet has greatly improved the accessibility, efficiency, and personalization of elderly care services. For example, remote medical care, online health consulting, virtual socializing, and smart living assistance devices have not only improved the quality of life for older people [3–4], but also provided new methods for disease prevention and health management [5–6]. Secondly, the internet has promoted innovation in elderly care service models by providing a more open and flexible service platform. Traditional elderly care services are often limited to home or institutional care, but technological advancements have made “internet + elderly care” models possible [7]. These models pay more attention to personalized elderly care needs, thus improving the quality and satisfaction of services. Finally, the internet has improved the overall efficiency of elderly care services by integrating resources and optimizing management [8–9]. Elderly care service platforms can achieve optimal resource allocation [10], smart technology can improve the accuracy of nursing and services, and data analysis can provide a basis for quality improvement in elderly care services [11–12].

The impact of elderly care services on internet innovation is also significant. The diversification and specialization of elderly care service demands have driven continuous optimization of internet technology, leading to advancements in the development of smart devices and personalized service customization [13–14]. This impact has not only promoted innovation in elderly care-related technologies and products, but also facilitated cross-industry collaboration and integration [15]. On the one hand, in order to meet the increasingly complex and detailed health management needs of older people, companies continuously innovate and develop a range of health management applications [16], smart wearables, and integrated systems tailored for the elderly [17]. On the other hand, the integration of resources and capabilities from various entities [18] such as enterprises, communities, governments, and society [19] through internet platforms has created comprehensive and multifunctional elderly care service systems [20]. This not only enhances the quality and efficiency of services [21] but also provides new business models and development opportunities for companies [22].

To summarize, while scholars have been quite prolific in independent research on these two fields, there is a relative dearth of research on how they can be mutually reinforcing and co-developing. This deficiency not only restricts the comprehensive understanding of the integration and development of the two industries, but also hinders the in-depth advancement of related policy formulation and practical application. At present, the analysis of the integration of the Internet and senior care services still stops at the stage of theoretical exploration of the integration of concepts and technologies, and scholars have not explored the actual coupling of the Internet and senior care services from the perspective of data. Therefore, this study aims to comprehensively and deeply explore the coupling and coordinated development level between the Internet and senior care services and its influencing factors by systematically sorting out the contents of previous studies, and utilizing the entropy value method, the coupling coordination degree model, the Tobit model and other empirical analysis methods. It is expected to provide researchers in related fields with new research perspectives and methodological references, and to provide policy makers and practitioners with strong scientific basis and practical guidance.

## Data collection

### Data sources

The term “smart aging” only appeared in China in 2013, and scholars only began to study how to apply Internet technology to the field of aging. China’s research on “Internet + Elderly” was conducted relatively late, in order to ensure the authenticity, scientifity and quality control of the data source, the final selection of 2012 as the base period, so as to examine the overall trend of the 2012–2021 period, the latest year’s data is not yet available. As China’s senior care services started relatively late, the different levels of economic development, resource allocation and provincial conditions in each province and city have led to varying levels of Internet senior care development around the country. The provinces have not yet established a network platform for information interoperability and resource sharing, which leads to the inability of the provinces to make statistics in the field of elderly care. Therefore, this paper studies the measurement of the level of coupled and coordinated development of Internet and senior care services in China at the macro level and analyzes the influencing factors. The original data used in this study mainly comes from the “China Statistical Yearbook” and “Statistical Bulletin of National Economic and Social Development of China” and “China Internet Development Statistical Report” from 2012 to 2021, as well as publicly available data from government websites. For some missing data, the mean value method was used to complete the dataset. The raw data are secondary data, all from existing databases.

### Selection of evaluation indicator variables

In order to deeply explore the level of coupled and coordinated development of the Internet and elderly services in China, this paper constructs a comprehensive evaluation index system. The system covers two major subsystems, namely, the Internet and elderly services, with the former further subdivided into two parts, namely, Internet infrastructure and Internet popularization capacity, and the latter further subdivided into two parts, namely, supply capacity of elderly services and basic protection of elderly services. The selection of these indicators aims to comprehensively and objectively assess the degree of integration of the two systems and provide a scientific basis for policy formulation and practical operation.

As the cornerstone of the coupled and coordinated development of the Internet and senior care services, the degree of perfection of the Internet infrastructure is crucial. The four selected indicators - the number of Internet broadband access ports, the number of CN domain names, the number of IPV4 addresses and the number of web pages - can comprehensively reflect the technical level and resource richness of the Internet infrastructure and provide stable and efficient network support for senior care services. Specifically, the number of Internet broadband access ports [23] reflects the level of Internet access hardware facilities to provide stable network support for elderly services. The number of CN domain names reflects the abundance of Internet resources, which helps senior care services to realize localized Internet access, and the number of IPV4 addresses indicates the scale of Internet users, which ensures the prerequisite for senior care services to access the Internet. The number of web pages [24] showcases Internet information resources to provide multiple information and service options for senior care.

Internet penetration capacity is an important indicator of the breadth and depth of the integration of the Internet with elderly services. The five selected indicators - cell phone penetration rate, number of cell phone subscribers, number of people with Internet access, Internet penetration rate and number of Internet broadband access subscribers - not only reflect the public’s acceptance of the Internet, but also the interaction between the elderly and the Internet, which is important for enhancing the convenience of senior care services and the quality of life for the elderly It is of great significance. Specifically, the cell phone penetration rate [25] and the number of cell phone subscribers reveal the coverage of Internet services, providing more elderly people with mobile Internet services. The Internet penetration rate [26], the number of people with Internet access and the number of Internet broadband access subscribers [27] reflect the extent of the public’s use of the Internet, enhancing the accessibility and convenience of elderly services.

Good supply can generate a huge amount of demand, and increased consumer demand can drive the convergence of the Internet and senior care services. The development of the integration of the Internet and the elderly is centrally embodied in a new type of elderly care service based on institutional elderly care and family elderly care, incorporating modern information technology such as artificial intelligence, breaking through the geographic, time, quality, and efficiency constraints on the supply of elderly care services, and meeting the growing demand for elderly care services in the community. The indicator of the number of nursing beds per 1,000 elderly population reflects the supply capacity of nursing services in general; indicators such as the number of nursing institutions [28] and the number of beds in nursing institutions [29] can reflect the current status of the supply of nursing services in nursing institutions; and the indicators of community service centers and community service stations reflect the current status of the supply of home-based nursing services based on the community.

China’s ever-improving basic old-age insurance system protects the basic livelihood of elderly urban and rural residents through the sharing of responsibilities among individuals, enterprises, collectives and the Government. The number of people participating in basic old-age insurance at the end of the year [30] is therefore included as an evaluation indicator. With the establishment of a sound temporary assistance system in China, some rural populations and urban low-income families in especially difficult circumstances are also able to receive temporary assistance from the Government. Therefore, the number of people enjoying the minimum subsistence guarantee [31] is included in the evaluation indicators. The Internet-based pension services include medical services, which may increase the demand for pension services when the medical services of the elderly are satisfied, so this paper uses the indicator of the number of health technicians [32] to represent the input of medical personnel. The detailed descriptions of the variables can be found in Table 1.

**Table 1:** Evaluation index system for the coordinated development of internet and elderly care services

Estate	Composite indicators	Evaluation indicators	Unit	Characteristic
Internet business	Internet infrastructure	Number of Internet broadband access ports	ten thousand	+
		Number of CN domain names	ten thousand	+
		Number of IPV4 addresses	ten thousand	+
		Number of pages	ten thousand	+
	Internet penetration capacity	Cell phone penetration rate	departments/100 persons	+
		Mobile telephone subscriber	ten thousand	+
		Internet access	ten thousand people	+
		Internet penetration	%	+
		Number of Internet broadband access subscribers	ten thousand	+
Pensions industry	Supply capacity of elderly services	Number of elderly care institutions	ten thousand	+
		Number of beds in elderly care institutions	ten thousand	+
		Number of beds per 1,000 elderly population	sheets/thousand	+
		Community service centers	ten thousand	+
		Community service stations	ten thousand	+
	Basic guarantees for old-age services	Number of persons covered by the minimum subsistence allowance	ten thousand people	-
		Number of participants in basic pension insurance at the end of the year	ten thousand people	+
		Number of health technicians	ten thousand people	+

### Influencing factor variable selection

This study hypothesizes that government management ability, financial support, science and technology investment, innovation level and informatization level will all have an impact on the development of the integration of the Internet and senior care services. The explanatory variable is the degree of coupling and coordination between the Internet and senior care services, and the explanatory variables are government management ability, financial support, scientific and technological input, innovation level and informatization level, which are measured by the indicators of total investment in fixed assets of the whole society, incremental increase in the scale of social financing, the proportion of scientific and technological expenditure to financial expenditure, the number of patents granted, and Internet broadband access users, respectively.

The government’s management capacity [33] refers to the government’s ability to formulate and supervise policies, regulate and manage the market order, establish a big data information platform, and promote the precise supply of elderly services. The development of the “Internet + elderly” service model requires top-level design and guidance. Therefore, the total investment in fixed assets of the whole society is chosen to measure the government’s management capacity.

Financial support [6] refers to government support for senior care services in accordance with relevant policies, including support from government departments in terms of land, taxes, fee waivers and financial policies, as well as the launch of senior care-related financial products by financial institutions. Since most of the projects involved in senior care services are micro-profit projects, subsidies and honors are needed to support Internet senior care enterprises and increase their attractiveness to enter the field of senior care services. Therefore, the incremental social financing scale is chosen to measure the strength of financial support.

Scientific and technological investment [34] refers to the investment of enterprises in research and development, technological innovation and the introduction of new technologies. The development of pension needs to use the Internet technology and platform, the individual pension “island” link up, form “Internet + pension” new network, change the way of pension resource allocation, realize the traditional pension industry of the new generation upgrade. Therefore, the proportion of science and technology expenditures in financial expenditures is chosen to measure the level of scientific and technological investment.

The level of innovation [35] refers to a series of activities of transforming scientific and technological innovations and applying them to social production so as to obtain economic and social benefits. Innovation can activate the internal power of Internet pension development, patent research and development to a certain extent represents the level of scientific and technological innovation, so this paper selects the number of patents authorized to represent the level of innovation [36].

The level of informatization [37] refers to the degree of development of scientific and technological means based on modern science and technology, such as interconnected communication technology and big data network, to classify and integrate specific information for use by specific groups of people according to their needs in a specific region, and represents the modernization process of a region. Under the wave of the Internet, the level of informatization of elderly services is still relatively low, and there is a lack of informatized elderly service programs for the specific needs of the elderly, and the scope of collecting and managing information on the lives of the elderly is relatively narrow. Therefore, Internet broadband access users are selected to represent the level of informatization. The detailed descriptions of the variables can be found in Table 2.

**Table 2:** Tobit regression model indicator system

Variable type	Variable name	Variable symbol	Description of variables	Unit
Explained variable	Degree of coupling coordination	D	Results of the coupling coordination degree modeling	—
Explanatory variable	Government management capacity	GOV	Total investment in fixed assets of the whole society	billions
	Financial support	FIN	Increase in the scale of social financing	billions
	Scientific and technological input	TECH	Science and technology expenditures as a share of fiscal expenditures	%
	Innovation level	INV	Number of patents granted	piece
	Informatization level	INFO	Internet broadband access subscribers	ten thousand

The dependent variable in this study is the coupling coordination degree between the internet and elderly care services. According to research conventions, it is divided into “0.0-0.1 extremely disordered, 0.1–0.2 severely disordered, 0.2–0.3 moderately disordered, 0.3–0.4 slightly disordered, 0.4–0.5 slightly coordinated, 0.5–0.6 mildly coordinated, 0.6–0.7 moderately coordinated, 0.7–0.8 highly coordinated, 0.8–0.9 extremely coordinated, 0.9-1.0 exceptionally coordinated” (as shown in Table 3).

**Table 3:** Classification standards of coupling coordination degree

Coupling Degree (D-value)	Coordination Type	Coupling Degree (D-value)	Coordination Type
0.0 ~ 0.1 0.1 ~ 0.2 0.2 ~ 0.3 0.3 ~ 0.4 0.4 ~ 0.5	Extremely Disordered Severely Disordered Moderately Disordered Slightly Disordered Slightly Disordered	0.5 ~ 0.6 0.6 ~ 0.7 0.7 ~ 0.8 0.8 ~ 0.9 0.9 ~ 1.0	Slightly Coordinated Mildly Coordinated Moderately Coordinated Highly Coordinated Exceptionally Coordinated

## Model selection

This study explores the coordinated and harmonious development of the internet and elderly care services, as well as their main influencing factors. The research is conducted in three steps. The first step applies the entropy method to determine the indicator weights. Entropy value method is an objective assignment method based on the principle of information entropy, which determines the weight of each indicator by calculating the information entropy of the indicator, thus avoiding the bias that may be brought about by the subjective assignment method. In this study, the entropy method is used to determine the weights of each evaluation index of Internet and senior care services to ensure the objectivity and accuracy of the evaluation. The main reasons why the article adopts the entropy value method are: firstly, there are many complex factors affecting the development level of the Internet and senior care services, which can be analyzed by using the entropy value method of empowerment; secondly, the entropy value method has a higher credibility compared with the subjective empowerment method, which calculates the weights according to the amount of information provided by the data of each indicator in the evaluation system, and is not affected by external factors. The specific steps are as follows:

Standardization of data:

1 $${{\text{y}}_{{\text{ij}}}} = \frac{{{{\text{y}}_{\text{j}}}\left( {\text{t}} \right) - {{\text{y}}_{{\text{min}}}}}}{{{{\text{y}}_{{\text{max}}}} - {{\text{y}}_{{\text{min}}}}}}$$

Calculate the weights of the indicators:

2 $${{{\text{P}}_{{\text{ij}}}} = \frac{{{\text{y}}_{{\text{ij}}}^\prime }}{{\sum _{{\text{i}} = 1}^{\text{m}}{\text{y}}_{{\text{ij}}}^\prime }}}$$

Calculate the entropy value of the indicators:

3 $${e_j} = - \frac{1}{{{\text{ln}}{\mkern 1mu} m}}\sum\limits_{i = 1}^m {{p_{ij}}} {\text{ln}}{\mkern 1mu} {p_{ij}}$$

Calculate the coefficient of variation:

4 $${{\text{g}}_{\text{j}}} = 1 - {{\text{e}}_{\text{j}}}$$

Determine the weights of the indicators:

5 $${{\text{w}}_{\text{j}}} = \frac{{{{\text{g}}_{\text{j}}}}}{{\sum\limits_{{\text{j}} = 1}^{\text{n}} {{{\text{g}}_{\text{j}}}} }}$$

The second step involves selecting a Coupling Coordination Model to measure the level of coupling and coordination in various indicators between the Internet and elderly care services. The coupled coordination degree model is an important tool for measuring the degree of mutual influence and interaction between two or more systems [26].It can not only reflect the correlation between systems, but also reveal the level of coordinated development between systems. We choose the coupled coordination degree model to study the coordinated development between the Internet and senior care services, mainly because it can comprehensively consider multiple aspects and multiple indicators of the two systems, so as to arrive at a comprehensive evaluation result. Compared with other models, the coupled coordination degree model pays more attention to the intrinsic connection and dynamic changes between systems, and can more accurately reflect the level of coordinated development between systems [38].In addition, the model has the advantages of simple operation and intuitive results, which facilitates our in-depth analysis and research. The specific steps are as follows:

Calculate the comprehensive evaluation index:

6 $${{\text{F}}_{\text{i}}} = \sum\limits_{{\text{j}} = 1}^{\text{n}} {{{\text{w}}_{\text{j}}}} {\text{y}}_{{\text{ij}}}^\prime $$

Calculate the coupling degree:

7 $${C = \frac{{2\sqrt {{F_x} \times {F_y}} }}{{{F_x} + {F_y}}}}$$

Calculate the coupling coordination degree:

8 $${{\text{D}} = \sqrt {{\text{C}} \times \left( {\alpha {{\text{F}}_{\text{x}}} + \beta {{\text{F}}_{\text{y}}}} \right)} }$$

In the equations, C represents the coupling degree between the two systems, D represents the coupling coordination level between the two systems, ${\text{F}}_{\text{x}}$and${\text{F}}_{\text{y}}$represent the development levels of the Internet and elderly care services respectively. α represents the relative importance of the Internet system in integration, and β represents the relative importance of the elderly care services system. This study assumes that the Internet and elderly care services are equally important, thus α = β = 0.5.

The third step involves the use of the Tobit model to measure the main influencing factors of the coupling coordination development between the Internet and elderly care services. The Tobit model, originally proposed by the American economist Tobit in 1958, skillfully blends traditional multiple regression analysis with the Probit model to construct a regression model that is particularly well suited for dealing with restricted dependent variables [39].In this study, given that the value of the coupled coordination between the Internet and senior care services is limited to the range of (0, 1), it clearly belongs to the restricted variables. If the conventional least squares method is used for the analysis, it will inevitably lead to estimation bias. However, the Tobit model can effectively solve the problems caused by the restricted dependent variable and provide us with more accurate parameter estimation and inference. The specific formula is as follows:

9 $${{\text{D}}_{\text{i}}}{\text{ = }}\alpha {\mkern 1mu} {\text{ + }}{\mkern 1mu} {\beta _{\text{1}}}{\text{GOV}}{\mkern 1mu} {\text{ + }}{\mkern 1mu} {\beta _{\text{2}}}{\text{FIN}}{\mkern 1mu} {\text{ + }}{\mkern 1mu} {\beta _{\text{3}}}{\text{TECH}}{\mkern 1mu} {\text{ + }}{\mkern 1mu} {\beta _{\text{4}}}{\text{INV}}{\mkern 1mu} {\text{ + }}{\mkern 1mu} {\beta _{\text{5}}}{\text{INFO}}{\mkern 1mu} {\text{ + }}{\varepsilon _{\text{i}}}$$

In the formula, ${\text{D}}_{\text{i}}$ represents the coupling coordination degree between the Internet and elderly care services in year i. ${\alpha }$ is the constant term, ${{\beta }}_{1}$ to ${{\beta }}_{5}$ are the coefficients of the respective variables, and ${{\upepsilon }}_{\text{i}}$ is the random error term.

## Results

### Overall development level of coordinated coupling between internet and elderly care services

On the whole, the coupling coordination between the Internet and senior care services is in the primary stage, and the coupling coordination of the two is on the rise, and there is still a lot of room for development in the future. As can be seen from Table 4, the mean value of the coupling coordination degree of the Internet and senior care service is 0.6310, indicating that the coupling coordination grade of the Internet and senior care service is low, in a mild coordination state. 2012–2021 China’s coupling coordination degree of the Internet and senior care service rises from 0.1413 to 0.9830, and during the period of time presents an annual incremental state, indicating that the coupling coordination of the Internet and senior care service is in an upward trend.

### Analysis of the time dimension of coordinated coupling between internet and elderly care services

**Fig. 1:**
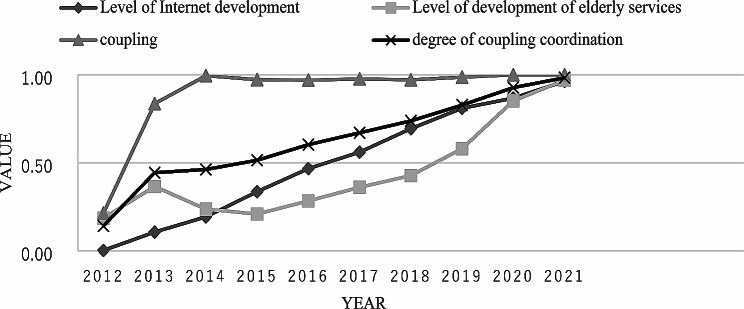
Coupling Coordination Development Level of Internet and Elderly Care Services in China

From a time perspective, the coordinated development of the internet and elderly care services can be divided into three stages: disorderly decline - transitional harmony - coordinated development. From Fig. 1, we can see that the first stage is 2012, and the coupling coordination degree is 0.1413, which is in the stage of serious dissonance. The second stage is 2013–2015, when the coupling harmonization increased from 0.4438 to 0.5145, which is in the transitional reconciliation stage. In the third stage, 2016–2021, the coupling coordination degree increases from 0.6031 to 0.9830, which is in the stage of coordinated development.

**Table 4:** Composite development index and coupling coordination degree of internet and elderly care services in China

Year	Internet Composite Index	Elderly Care Service Composite Index	Coupling Degree	Coordination Index	Coupling Coordination Degree	Coupling Coordination Level
2012	0.0021	0.1849	0.2134	0.0935	0.1413	Severely Disordered
2013	0.1063	0.3651	0.8357	0.2357	0.4438	Slightly Disordered
2014	0.1927	0.2364	0.9947	0.2145	0.4620	Slightly Disordered
2015	0.3358	0.2087	0.9723	0.2722	0.5145	Slightly Coordinated
2016	0.4669	0.2833	0.9695	0.3751	0.6031	Mildly Coordinated
2017	0.5604	0.3608	0.9762	0.4606	0.6706	Mildly Coordinated
2018	0.6937	0.4273	0.9713	0.5605	0.7379	Moderately Coordinated
2019	0.8099	0.5803	0.9862	0.6951	0.8279	Highly Coordinated
2020	0.8675	0.8516	0.9999	0.8595	0.9271	Exceptionally Coordinated
2021	0.9628	0.9698	0.9999	0.9663	0.9830	Exceptionally Coordinated
Average Values	0.4998	0.4468	0.8919	0.4733	0.6310	Mildly Coordinated

### Analysis of the influencing factors of coordinated coupling between internet and elderly care services

Among the influencing factors of the coupling and coordination degree of the Internet and senior care services, the government management ability has a more positive influence on the integration and development of the Internet and senior care services, financial support, scientific and technological investment and innovation level play a mild pulling role, while the level of informatization to a certain extent restricts the level of integration of the Internet and senior care services. Table 5 shows that the regression coefficient of government management ability is 1.448, and the P-value is 0.000, indicating that government management ability passes the test of significance at the 1% level, and plays a significant positive role in the coupled and coordinated development of the Internet and senior care services. The coefficient of financial support is 0.209, with a P value of 0.002, indicating that financial support passes the test of significance at the 1% level and plays a positive role in the coupling and coordination of the development of the Internet and senior care services. The coefficient of innovation level is 0.222, P value is 0.000, indicating that the level of innovation passes the test of significance at 1% level, and plays a significant positive role in the coupling and coordination of the development of the Internet and senior care services. The coefficient of science and technology input variable is 0.149, with a P value of 0.015, indicating that the science and technology input passes the significance test at the 5% level and has a positive effect on the coupling and coordination of the development of the Internet and senior care services. The coefficient of the informatization level variable is -0.571, with a P value of 0.000, indicating that the level of informatization passes the significance test at the 1% level, which indicates that the level of informatization will have a negative effect on the coupled coordination of the two systems.

**Table 5:** Tobit regression model results

Variables	Regression coefficient	Standard error	Statistic	*P*-value	Coefficient 95% confidence interval	
Total investment in fixed assets of the whole society	1.448***	0.174	8.331	0.000	1.789	1.107
Increase in the scale of social financing	0.209***	0.067	3.143	0.002	0.340	0.079
Science and technology expenditures as a share of fiscal expenditures	0.149**	0.062	2.423	0.015	0.270	0.028
Number of patents granted	0.222***	0.061	3.618	0.000	0.342	0.102
Internet broadband access subscribers	-0.571***	0.140	-4.064	0.000	-0.295	-0.846

Note: *, **, and *** indicate significance at the 10%, 5%, and 1% statistical levels

## Discussion

### Key finding

The mean value of the coupling coordination degree of the Internet and senior care service is 0.6310, indicating that the coupling coordination level of the Internet and senior care service is low and in a mildly coordinated state. The coupling coordination degree of Internet and senior care service in China increases from 0.1413 to 0.9830 from 2012 to 2021, and the period shows an annual increment, indicating that the coupling coordination of Internet and senior care service is on an upward trend. China’s Internet and senior care service integration development has been developed for a long time, and many achievements have been made, but the level of integration development of the two is still in a mildly coordinated state. In the face of the diverse needs of elderly services in the future, we promote the continuous improvement of the level of integrated development of the Internet and elderly services, which is conducive to the use of information technology, improves the quality of elderly services, smooths the channels of exchange of information on elderly services, provides a good supply of resources for elderly services, and promotes the diversified development of elderly services.

The coordinated development of the Internet and senior care services has gone through three stages of dysfunctional decline-transitional reconciliation-coordinated development, indicating that the coupled and coordinated development of the Internet and senior care services has gained momentum and is gradually converging towards coupled coordination. By understanding the development trend of the Internet and senior care services, it not only fills the gaps of the existing research, but also helps to reveal the development status of the coupling and coordination of the Internet and senior care services, and finds that Internet senior care is the business model that really meets the needs of the elderly, and provides theoretical support and decision-making references for the planning and management of the multi-party main body.

In terms of influencing factors, government management ability has a more positive impact on the integrated development of the Internet and senior care services, financial support, scientific and technological investment and innovation level play a mild pulling role, while the level of informatization restricts the level of integration of the Internet and senior care services to a certain extent. Among them, the role of government management capacity is the strongest, which indicates that through the state issued policy documents on the integration and development of the Internet and senior care services, it is conducive to the establishment of the standard system of the Internet senior care industry, standardize the market order, and guide the synchronous benign development of the Internet and senior care services. It is also conducive to the integration of various types of service resources, improve service trust and ensure service quality, and meet the personalized and diversified needs of the elderly. Instead, the level of informatization will have a negative impact on the coupling and coordination of the two systems, because the elderly are affected by their own limited economic conditions, low cultural level, limited acceptance of new things and other factors, resulting in their inability and unwillingness to use the service platforms and smart devices. Due to the independence of the information platforms of various organizations in Internet aging, there are certain differences in the data of various platforms for the elderly, which affects data docking and is not conducive to the realization of data interconnection. In addition, enterprises lack an aging perspective when designing smart products, failing to fully understand the use scenarios and real-life confusion of the elderly, and focusing more on technology and performance, which is not conducive to the elderly’s proficiency in the use of smart devices, thus making the problem of the digital divide among the elderly increasingly prominent and restricting the development of the Internet-based elderly care model.

### Strengths

This study chooses to take the Internet and senior care services as the research object to explore the coupled and coordinated relationship between the Internet and senior care services. The possible innovations are: first, although there is a considerable amount of literature studying the Internet and senior care services separately, there are relatively few studies combining the two and focusing on their coordinated development, so this paper attempts to do so. Secondly, most of the existing studies on the Internet and senior care services are mainly theoretical, with limited quantitative analysis of the coordinated development of the two, and a lack of analysis of the reality of the coordinated development of the Internet and senior care services, which makes it difficult to provide policy recommendations. In view of this, this study is based on the panel data of China’s Internet and senior care services from 2012 to 2021, constructs an evaluation index system for the integrated development of the Internet and senior care services, measures the level of integrated development of the Internet and senior care services by using the entropy method and the coupled coordination degree model, analyzes the integration posture and evolution law, and puts forward the strategic suggestions for the integrated development of the Internet and senior care services based on this, with a view to promote the synergistic development of Internet and senior care services.

### Limitations

It is worth noting that this study may still have some limitations. First, at the time scale level, the data used in this study are mainly from existing governmental public statistical yearbooks, and given the time-sensitive nature of data updates, the data collected in this study are only available up to 2021, and data for the most recent year are not yet available, but this does not affect the conclusions of this study, which are to be further advanced in the future. Second, at the spatial scale level, this study examines national macro-level data from China for the decade from 2012 to 2021, and has not yet covered the micro-levels such as the provincial, municipal, and county levels, which also makes the depth of this paper’s research still insufficient. Third, the evaluation indicators need to be optimized. This study uses national-level data to construct an evaluation index system for the Internet and elderly services, but there are still some deficiencies in the selection of indicators and the construction of evaluation models.

Future research direction, the research on the coupling and coordination of the Internet and elderly services is both a systematic and hierarchical topic and a long period of cumulative research process. In the follow-up research, the research scale can be sunk to the meso- and micro-level, combined with typical regions to conduct more detailed research, and promote the in-depth development of research in this field forward.

## Conclusion

The purpose of this study is to explore the coordinated development of the internet and elderly care services in China. By collecting publicly available data from 2012 to 2021, the study utilizes entropy method, coupling coordination degree model, and Tobit model for research analysis. The main findings are as follows:(1) The overall level of coordination between the internet and elderly care services in China shows an upward trend, with a mean value of 0.63, indicating a primary level of coordination. The coupling coordination degree of Internet and senior care service in China increases from 0.1413 to 0.9830 from 2012 to 2021, and the period shows an annual increment, indicating that the coupling coordination of Internet and senior care service is on an upward trend. It shows that at present, the integration of Internet and pension services in China is still in the primary stage, to realize the wisdom of the elderly, there is still a long way to go in the future, and there are still many problems that need to be solved.

(2) In terms of time scale, the coordinated development of the internet and elderly care services in China has gone through three stages: “disorderly decline - transitional harmony - coordinated development.” It shows that the level of coupling coordination between the two is a continuous upward trend, coupling coordination level from dysfunctional decline to coordinated development, coupling coordination development momentum is good, gradually converging to synergistic, but did not achieve a high degree of coordinated development, coupling coordinated development is still a large space for improvement.

(3) The degree of coupling coordination is affected by a variety of factors, and there are significant differences in the effects of different factors. Government management ability, financial support, scientific and technological investment and innovation level have a more positive and positive impact on the development of the integration of the Internet and senior care services. Among them, the role of government management ability is the strongest, the reason is that senior care service has the attribute of quasi-public goods, and its development needs to rely on government policy support. And the level of informatization restricts, to a certain extent, the improvement of the level of integration between the Internet and senior care services. In the future, the need to further expand the effect of the role of each factor is an important direction to promote the coordinated development of the Internet and senior care services. In conclusion, the integration of the Internet and senior care services is an inevitable choice for solving the current aging problem in China. Although the integration of the two is still in the primary stage, the coupling and coordination of the two is on the rise, so China’s Internet-based elderly care service has the potential to be more coordinated and sustainable in the future.

Based on the conclusions of this study, the following recommendations are proposed: (1) Deepening cognition: a comprehensive understanding of the current status and development space of the coupling coordination degree between the Internet and senior care services in China. Given that the mean value of the coupling coordination between the Internet and senior care services in China is 0.6310, it reveals that the two are currently in a mildly coordinated state, showing that there is still significant potential for integration between the two. This value not only emphasizes the importance of deepening the understanding of the current state of coordination, but also reminds us that this integration is a dynamic and continuous process that requires policymakers, researchers, scholars, and practitioners to pay attention to and make efforts to promote its development to a deeper level. Policymakers need to make policy adjustments and innovative practices based on the current state of this harmonization, taking into account actual needs, in order to promote the deeper integration of the two.

(2) Trend-oriented: accurately grasp the dynamic trend of the coupling and coordinated development of China’s Internet and senior care services, and promote the stage of leaping up. China’s Internet and senior care services coupling and coordinated development has experienced from “disorder and decline” to “transition and reconciliation”, and then to the current “coordinated development” stage, and shows an overall rising Trend. This development not only reflects the gradual maturity of the integration of the two, but also provides clear guidelines for the future direction of development. Policymakers should closely follow this development trend and promote the coordinated development of coupling from the current stage to a higher level through precise policy guidance and market mechanisms. In the process of promoting the stage leap, the characteristics and challenges of each stage should be analyzed in detail, and relevant policies and measures should be formulated and implemented in a targeted manner. For example, at the stage of dislocation and decline, there is a need to focus on resolving the problems of uneven distribution of resources and insufficient supply of services; at the stage of transition and reconciliation, there is a need to focus on the innovation and transformation of service models; and at the stage of coordinated development, there is a need to pay more attention to the enhancement of the quality and efficiency of services. At the same time, the implementation of these policies and measures needs to be synchronized with market demands and changes to ensure the continuity and stability of coupled and coordinated development.

(3) Factor Optimization: Fully consider the influencing factors to achieve the optimal allocation of policies and resources. Factors such as government management ability, financial support, technology investment and innovation level play a key role in promoting the coupled and coordinated development of Internet and elderly care services in China. Policymakers and resource allocators need to pay full attention to these influencing factors and optimize them in policy formulation and resource allocation. For example, it is important to enhance the government’s ability to collaborate across sectors and synergize policies, strengthen financial support for the senior care industry, increase the investment of science and technology in senior care, and stimulate the innovation vitality of enterprises and social organizations. Although the level of informatization negatively affects the coupled and coordinated development to a certain extent, we should still pay attention to the importance of informatization construction, and enhance the quality and efficiency of senior care services by promoting data sharing, information security standard-setting, and the wide application of information technology in senior care services.

### Electronic supplementary material

Below is the link to the electronic supplementary material.

Supplementary Material 1

## Data Availability

Data are provided in manuscripts or supplementary information documents.
